# Risk of acute kidney injury and survival in patients treated with Metformin: an observational cohort study

**DOI:** 10.1186/s12882-017-0579-5

**Published:** 2017-05-19

**Authors:** Samira Bell, Bassam Farran, Stuart McGurnaghan, Rory J. McCrimmon, Graham P Leese, John R Petrie, Paul McKeigue, Naveed Sattar, Sarah Wild, John McKnight, Robert Lindsay, Helen M. Colhoun, Helen Looker

**Affiliations:** 1 Renal Unit, Ninewells Hospital, Dundee, DD1 9SY UK; 2 Division of Population Health Sciences, School of Medicine, University of Dundee, Dundee, UK; 3 Institute of Genetics and Molecular Medicine University of Edinburgh, Edinburgh, UK; 4 Division of Molecular & Clinical Medicine, School of Medicine, University of Dundee, Dundee, UK; 5 Department of Medicine, University of Dundee, Dundee, UK; 6 Institute of Cardiovascular and Medical Sciences, BHF Glasgow Cardiovascular Research Centre, University of Glasgow, Glasgow, UK; 7 Centre for Population Health Sciences, University of Edinburgh Medical School, Edinburgh, UK; 8 Department of Medicine, Western General Hospital, Edinburgh, UK

**Keywords:** Acute kidney injury, Diabetes, Epidemiology, Metformin, Survival

## Abstract

**Background:**

Whether metformin precipitates lactic acidosis in patients with chronic kidney disease (CKD) remains under debate. We examined whether metformin use was associated with an increased risk of acute kidney injury (AKI) as a proxy for lactic acidosis and whether survival among those with AKI varied by metformin exposure.

**Methods:**

All individuals with type 2 diabetes and available prescribing data between 2004 and 2013 in Tayside, Scotland were included. The electronic health record for diabetes which includes issued prescriptions was linked to laboratory biochemistry, hospital admission, death register and Scottish Renal Registry data. AKI events were defined using the Kidney Disease Improving Global Outcomes criteria with a rise in serum creatinine of at least  26.5 μmol/l or a rise of greater than 150% from baseline for all hospital admissions. Cox Regression Analyses were used to examine whether person-time periods in which current metformin exposure occurred were associated with an increased rate of first AKI compared to unexposed periods. Cox regression was also used to compare 28 day survival rates following first AKI events in those exposed to metformin versus those not exposed.

**Results:**

Twenty-five thousand one-hundred fourty-eight patients were included with a total person-time of 126,904 person years. 4944 (19.7%) people had at least one episode of AKI during the study period. There were 32.4 cases of first AKI/1000pyrs in current metformin exposed person-time periods compared to 44.9 cases/1000pyrs in unexposed periods. After adjustment for age, sex, diabetes duration, calendar time, number of diabetes drugs and baseline renal function, current metformin use was not associated with AKI incidence, HR 0.94 (95% CI 0.87, 1.02, *p* = 0.15). Among those with incident AKI, being on metformin at admission was associated with a higher rate of survival at 28 days (HR 0.81, 95% CI 0.69, 0.94, *p* = 0.006) even after adjustment for age, sex, pre-admission eGFR, HbA_1c_ and diabetes duration.

**Conclusions:**

Contrary to common perceptions, we found no evidence that metformin increases incidence of AKI and was associated with higher 28 day survival following incident AKI.

## Background

Metformin is a biguanide which has been used as first line therapy for the treatment of type 2 diabetes in the United Kingdom since the 1960s. In addition to its effectiveness as a treatment for type 2 diabetes, there is increasing evidence of its beneficial cardiovascular effects [1–6]. There is, however, considerable debate regarding its use in patients with chronic kidney disease (CKD), defined as an estimated Glomerular Filtration Rate (eGFR) of less than 60 ml/min/1.73^2^. Guidelines pertaining to metformin prescribing and renal impairment vary. The United States Food and Drug Administration (FDA) previously stated that metformin was contraindicated in males with a serum creatinine above 132.6 μmol/l and females above 123.8 μmol/l or abnormal creatinine clearance [7]. However, following a recent review of the literature, these recommendations were revised to indicate that metformin can now be used safely in patients with mild renal impairment (eGFR 45- 60 ml/min/1.73^2^) and in some with moderate renal impairment (eGFR <45 ml/min/1.73^2^) [8]. The National Institute for Health and Care Excellence (NICE) in the UK allows prescribing of metformin in patients with an eGFR of less than 60 ml/min/1.73^2^ but recommends that the dose is reduced in patients with an eGFR of less than 45 ml/min/1.73^2^ (or serum creatinine of greater than 132.6 μmol/l) and metformin discontinued if eGFR is less than 30 or serum creatinine greater than 150.3 μmol/l [9]. Current contraindications therefore exclude its use in a large proportion of patients with CKD [10]. Following the FDA’s announcement, the European Medicines Agency (EMA) have also now announced that metformin-containing medicines can be used in patients with moderately reduced renal function (eGFR 30 to 59 ml/min/1.73^2^) [11].

The advent of a universally agreed serum creatinine based definition for AKI allows identification of even mild (Stage 1) AKI without relying on hospital coding systems [12] allowing assessment of renal safety in patients with CKD.

The present study therefore aimed to examine whether current metformin use was associated with an increased rate of AKI as a proxy for lactic acidosis compared to non-use and to establish whether metformin exposure affected survival in patients with AKI.

## Methods

### Study population

All adults over 18 between the 1st of January 2004 and 31st of December 2013 with type 2 diabetes and available prescribing data who were resident or died in the NHS Tayside region, Scotland formed the study cohort.

### Ethical statement

Anonymised record linkage was conducted according to Health Informatics Centre (HIC), University of Dundee [13] Standard Operating Procedure (SOP). The Tayside Research Ethics Committee does not require submission of individual studies that follow this SOP which is Caldicott Guardian approved.

### Data sources

Data were linked using HIC [13], University of Dundee. HIC enables anonymised health record linkage from the population of Tayside (400000), Scotland, using a unique identifying Community Health Index (CHI) number. Data were linked between the following datasets: Scottish Morbidity Record of hospital admissions (SMR01); laboratory results, medicines dispensed by community pharmacies, the Scottish Care Initiative-Diabetes Collaboration (SCI-DC) and the Scottish Renal Registry (SRR). SMR01 provides information on age, sex, postcode and admission and discharge dates. Creatinine measurements were obtained from the laboratory system. SCI-DC provided information on diabetes type and date of diagnosis. The number of dispensed prescriptions over the previous year prior to admission from community pharmacies, and exposure to medications that predispose to renal impairment (non-steroidal anti-inflammatory drugs (NSAIDs), Cox-2 inhibitors, ACE-inhibitors and Angiotensin-II receptor antagonists), were ascertained from dispensed prescribing data. This dataset comprises of all community dispensed prescribing from community pharmacies in the Tayside region of Scotland. Patients receiving chronic dialysis or post renal transplant were identified using the SRR.

### Outcomes

All hospital admissions during follow up were evaluated for the difference between the peak in-hospital and pre-admission serum creatinine. AKI severity was defined using the Kidney Disease Improving Global Outcomes (KDIGO) creatinine based criteria. AKI was defined as the rise in serum creatinine of at least 26.5 μmol/l or a serum creatinine of greater than 150% of baseline serum creatinine during admission [12]. Baseline creatinine was defined as the median of all creatinine measurements between 28 and 56 days prior to hospitalisation. Cases were excluded if a baseline creatinine was not measured. Estimated GFR was calculated using the Modification of Diet in Renal Disease (MDRD) 4 formula [14]. The primary outcome for the survival analysis was all-cause mortality within 28 days from the day of admission.

### Statistical methods

All type 2 diabetes patients who were at least 18 years old at diagnosis and were observable for drug prescriptions at some point during the study period were included. A patient was considered observable if they had records of drug prescriptions, with gaps of up to 180 days between prescriptions allowed. Their entry date was calculated as the latest of their date of diagnosis of diabetes, study entry date (1/1/2004), and their drug observability (between study start date and study end date (31/12/2013)). Follow-up was censored at the earliest of death, study end date, unobservability, or AKI event. The data was then split longitudinally into 28 day time slices, meaning each 28 days a patient contributed to the study results in one row in our data set. This allowed us to use time-updated covariates (eGFR, number of diabetes drugs being taken, diabetes duration, calendar time, ever/never metformin use, and HbA1c). If more than one measurement (such as eGFR or HbA_1c_) fell into the same time slice, the median was taken. Patients who contributed less than 28 days of follow-up were excluded. All observability stretches were extended by 90 days as drug observability ceases during hospitalisation. Drugs were identified by their Anatomical Therapeutic Chemical (ATC) codes, and drug unobservability meant a patient *had* not had a prescription of any drug for more than 180 days.

Baseline characteristics (ie those identified closest to study entry date) were displayed as mean and standard deviation (SD) or median and interquartile range (IQR) for continuous variables depending on distribution. Categorical variables were displayed as number and percentage.

### Survival analysis

Cox regression analyses, comparing AKI rates in person- time periods on metformin with AKI rates in person time periods not on metformin, were used to calculate hazard ratios (HR) and 95% confidence intervals adjusted for confounders. Confounders were selected based on literature, guidelines and clinical expertise and were age, sex, diabetes duration, baseline renal function, calendar time and number of diabetes drug classes [12, 15]. All analyses were carried out in R. Sensitivity analysis was performed using “ever” versus “never” exposure in place of current exposure where “ever” was defined as at least one prescription for metformin during the study period.

## Results

Between 1st of January 2004 and 31st of December 2013, there were 25,148 patients with Type 2 diabetes in the Tayside region of Scotland forming the study cohort. 14,622 of these patients were treated with metformin at some point during the study period and 10,526 patients were never on metformin during the study period. Total person-time was 126,904 person years (pyrs) of which 60,738 pyrs were exposed to metformin. Median duration of follow-up was 8.1 years IQR (5.2-10.0). Demographic characteristics of the study population at the study midpoint (1st July 2008) are described in Table 1. The analysis conducted here is a time updated analysis of rates in person-time periods (rather than *persons*) associated with metformin exposure compared to person-time periods without exposure therefore we show the characteristics of person-time periods associated with current metformin exposure compared to person-time periods without current exposure in Table 2. Metformin exposure periods occurred at younger ages, higher HbA_1c_, longer duration of diabetes and were associated with higher eGFR, *p* < 0.0002.

**Table 1:** Demographic characteristics of cohort at study midpoint 1st July 2008, *n* = 12,373

Age in years^a^	68.2 (59.2-75.9)
Sex (M) (%)	6636 (54)
Metformin Use (%)	5975 (48)
Diabetes Duration in years^a^	5.48 (2.50-9.91)
Number of diabetes drug classes prescribed (%)	
0	4187 (33.8)
1	4517 (36.5)
2	2943 (23.8)
3	671 (5.4)
4	53 (0.4)
5	2 (0.02)
eGFR category (%)	
>60	8926 (72.1)
30-59	2574 (20.8)
<29 ml/min/1.73^2^	180 (1.5)
Missing	693 (5.6)
HbA_1c_ %	7.0 (6.3 – 7.9)
(mmol/mol)	53 (45- 63)
ACE inhibitor or ARB Use (%)	5074 (41%)
NSAID or Cox-2 inhibitor use (%)	1160 (9.4%)

^a^Median & IQR

**Table 2:** Summary patient characteristics by metformin exposure status

	Metformin exposed periods	Metformin unexposed periods
	(60,738.1 years)	(66,165.9 years)
Age (Years) ^a^	65.7 (57.3 - 73.5)	70.2 (61.1 – 78.1)
HbA_1c_ %	7.3 (6.7- 8.3)	6.7 (6.2- 7.6)
(mmol/mol) ^a^	56 (50 – 67)	50 (44 - 60)
Diabetes Duration (years) ^a^	6.9 (3.7 – 11.2)	4.3 (1.8 – 8.9)
eGFR category (%)		
>60	44,830.6 (73.8)	39,332.4 (59.4)
30-60	12,575.1 (20.7)	18,775.3 (28.3)
<30 ml/min/1.73^2^	236.8 (0.4)	1592.7 (2.4)
ACE/ARB use (%)	25,572.6 (42)	21,827.7 (33)
NSAID use (%)	5967.4 (10)	6140.8 (9)

^a^Median & IQR

During follow-up, 4944 people had at least one episode of AKI during the study period. Of these AKI events, 4352 were Stage 1, 140 Stage 2 and 452 Stage 3 AKI events. Only person-time periods to the first event were included in analyses. The characteristics at entry into the study of those persons who had an event at any time during follow up versus those who did not are shown in Table 3. Patients who had any AKI event had lower eGFR at entry to the study than those who never developed an event (median 58 ml/min/1.73^2^ IQR 46-70 vs 73 ml/min/1.73^2^ IQR 61-88). AKI was strongly associated with pre-admission eGFR (Table 4).

**Table 3:** Characteristics of patients developing/ not developing AKI at entry into study

	AKI event during follow-up	No AKI event during follow-up
	(*n* = 4944)	(*n* = 20,204)
Age (Years) ^a^	71.7 (64.7 – 78.3)	63.4 (54.2 – 72.1)
Male Sex (%)	2694 (54.5)	11,031 (54.6)
HbA_1c_ %	7.2 (6.4- 8.3)	7.0 (7.4- 8.4)
(mmol/mol) ^a^	55 (46 – 67)	53 (45 - 68)
Diabetes Duration (years) ^a^	0 (0 – 3)	3.3 (0 – 9)
Number of diabetes drug classes (%)		
0	2583 (52)	14,212 (70)
1	1617 (33)	4471 (22)
2	706 (14)	1438 (7)
3	27 (0.5)	76 (0.4)
4	1	6
5	0	1
ACE/ARB use (%)	1505 (30)	4667 (23)
NSAID use (%)	474 (10)	2079 (10)

^a^Median & IQR

**Table 4:** Distribution of AKI incidence by baseline eGFR and AKI stage

	eGFR (ml/min/1.73^2^)								
AKI Stage	<30			30-60			>60		
	n	Person time (years)	Incidence rate (per 1000 person-years)	n	Person time (years)	Incidence rate (per 1000 person-years)	n	Person time (years)	Incidence rate (per 1000 person-years)
1	439	1829	240	2187	31,350	70	1717	84,163	20
2	16	1829	8.7	43	31,350	1.3	81	84,163	1.0
3	167	1829	91	142	31,350	4.5	129	84,163	1.5

Overall incidence of AKI was 39.0 cases/1000 pyrs. There were 32.4 cases of AKI/1000pyrs in person-time periods with current metformin use compared to 44.9 cases/1000pyrs during person-time periods in which metformin was not being used. Severity of diabetes was adjusted for by adjusting for diabetes duration and number of diabetes medicines. Current metformin use was associated with a lower AKI rate, HR 0.76 (95% CI 0.70, 0.82, *p* < 0.0001) after adjustment for sex, current age, current diabetes duration, and current number of diabetes drugs. When further adjusted for baseline renal function, current metformin use was no longer associated with any difference in AKI rate, HR 0.94 (95% CI 0.87, 1.02, *p* = 0.15). After including current or “ever” metformin use, person-time periods with current or “ever” exposure to metformin were associated with higher rates of AKI than person-time periods with no current or prior exposure; HR 1.13 (95%CI 1.05, 1.22, *p* = 0.001).

At study midpoint only 1.5% of people with an eGFR <30 were taking metformin compared to 72% of people with an eGFR >60. Clearly since current eGFR is a strong correlate of metformin exposure and predicts future AKI events, disentangling drug effects from allocation effects is difficult and negative confounding by allocation could explain the hazards ratio (HR) observed. In addition to adjusting for baseline eGFR in the Cox regression, we also examined whether, among strata of person-time periods defined by baseline eGFR, whether there was any evidence of an effect of metformin. We found no evidence of increased rates of AKI- even where baseline eGFR was <30, the rates of events in person-time periods exposed to metformin were not greater than those unexposed to metformin (Table 5). Furthermore we conducted a sensitivity analysis where we adjusted for lagged eGFR – i.e. for each time periods we included a time updated covariate which was eGFR 6 months prior to that time period as distinct from the baseline eGFR as that may have long preceded that person-time period. This analysis allows for a fuller adjustment of the effect of prevailing eGFR on the probability of metformin exposure. In this analysis the HR for current metformin exposure was 0.85 (95%CI 0.78, 0.92), *p* < 0.0001.

**Table 5:** Hazard ratios (95% CI) for AKI associated with metformin exposure compared to non-exposure by baseline eGFR

eGFR (ml/min/1.73^2^)	<30	30-60	>60
Number of events	88	733	681
HR (95% CI)	0.45 (0.10,1.91)	0.80 (0.66,1.0)	0.76 (0.62,0.95)
p	0.28	0.05	0.01

Note that since it could be argued that the probability of subsequent exposure to metformin (either for the first time or repeat prescription) may be altered when an AKI event has occurred, person- time was right censored at the first AKI event for this part of the analyses.

### Survival

Table 6 show the characteristics of those AKI events where there was current metformin exposure compared to events not associated with current exposure. Of patients with AKI, 844 died within 28 days of admission. Death rate was 23.5 (per 1000 pyears of follow-up): 7.9 in patients on metformin vs 15.6 in patients not on metformin per 1000 pyears. Conditional on the AKI event occurring, metformin use at admission was associated with a lower risk of death at 28 days (HR 0.81, 95% CI 0.69, 0.94, *p* = 0.006) even after adjustment for age, sex, pre-admission eGFR, HbA_1c_ and diabetes duration. Following further adjustment for use of ACE inhibitor or ARB, use of NSAIDs or Cox-2 inhibitors and number of prescribed medicines as a proxy for co-morbidity, this effect persists (HR 0.82, 95% CI 0.70, 0.95, *p* = 0.01). When examining longer term mortality to the end of 2013 in those who had experienced an AKI event, risk of death subsequent to an AKI event not on metformin was higher HR 1.32 (95%CI 1.23, 1.43, *p* < 0.0001) than risk of death with AKI treated with metformin HR 1.15 (95%CI 1.06, 1.25, *p* = 0.002) after adjustment for age, sex, baseline renal function, HbA_1c_ and diabetes duration.

**Table 6:** Characteristics of patients who developed AKI during follow-up (*n* = 4944)

	Metformin exposed AKI event	AKI event without concurrent metformin exposure
	(*n* = 1972)	(*n* = 2972)
Age (Years) ^a^	73.6 (65.7 – 79.4)	77.6 (70.4-83.7)
Male Sex (%)	1161 (58.9)	1533 (51.6)
HbA_1c_ %	7.2 (6.5- 8.3)	6.8 (6.2- 7.9)
(mmol/mol) ^a^	55 (48- 67)	51 (44- 63)
Diabetes Duration (years) ^a^	9.15 (5.11- 13.85)	7.50 (3.30-13.76)
Number of diabetes drug classes (%)		
0	13 (0.7)	1745 (58.7)
1	830 (42.1)	1049 (35.3)
2	897 (45.5)	162 (5.5)
3	212 (10.8)	16 (0.5)
4	19 (1.0)	0 (0.0)
5	1 (0.1)	0 (0.0)
CKD category (%)		
>60	941 (47.7)	986 (33.2)
30-59	896 (45.4)	1476 (49.37)
<29 ml/min/1.73^2^	130 (6.6)	492 (16.6)

^a^Median & IQR

## Discussion

In this large cohort of over 25,000 patients with Type 2 diabetes, we found no evidence of any adverse effect of current metformin use on AKI incidence or on survival following AKI across different eGFR strata. We have shown risk of AKI does not differ during person-time periods where there was no current metformin exposure compared to those in which there was current metformin exposure.

Strengths of our study include the large cohort size with high metformin use. We identified our cohort using the SCI-Diabetes system. This system is used to record all diabetes clinical care in Scotland thereby capturing 99% of patients with diabetes [16]. Furthermore, prescribing data was obtained from pharmacy dispensed prescriptions (covering all pharmacies in the Tayside region) rather than prescriptions alone increasing reliability that the medicines are actually being taken. AKI was defined using the KDIGO creatinine based criteria from laboratory data that are only available at regional level [12].

Observational pharmaco-epidemiology studies have to contend with various potential biases or confounders. The most difficult bias for this study is negative confounding by allocation wherein those with a lower eGFR are more likely to develop AKI but less likely to be exposed to metformin. We attempted to address this by adjustment for eGFR in a number of ways and by conducting analyses by different strata of eGFR. None of the analyses showed any suggestion of an adverse effect. Since there are unlikely to be randomised trials of allocating metformin to those with low eGFR to assess safety, this observational study provides useful reassurance that we cannot see any adverse safety signals in the data that would warrant a change to the current guidelines. If anything the data suggest that the current guidelines may be too stringent and could be relaxed further. We used multivariable analysis to adjust for important clinical confounders in view of the difference between the metformin users and the non-metformin users. Furthermore, our data may not be applicable to other populations as the Tayside region of Scotland lacks ethnic diversity and is predominantly of European ancestry. In addition, we were unable to use direct data on co-morbidity due to problems of under reporting [17]. We therefore used number of prescribed medications prior to admission which has been shown to correlate well with co-morbidity [18]. A further limitation of our study was that we were unable to account for over the counter NSAID use in our population.

Reported incidence rates of metformin associated lactic acidosis are in fact very low further supporting its more widespread use. A Cochrane review showed no cases of fatal or non-fatal lactic acidosis in 70, 490 patient- years of metformin use [19]. Incidence rates of MALA were 3.3 per 100,000 person-years in a nested case control analysis of 50,048 patients from the United Kingdom General Practice Research Database compared with 4.8 per 100,000 person-years among sulphonylurea users [20]. Several studies have shown use of metformin in patients with renal impairment thereby suggesting non-adherence to guidelines [21–25]. In a subgroup analysis of 6960 patients with eGFR 45-60 ml/min/1.73^2^ from a Swedish cohort of 51, 675 patients, there was a significantly lower risk of acidosis/ serious infection in patients on metformin and a slightly higher but insignificant risk in the subgroup of 2044 patients with eGFR of 30-45 ml/min/1.73^2^ [2]. A systematic review examining patients with CKD demonstrated the safety of metformin in patients with stable CKD (eGFR >30 ml/min/1.73^2^) including 150,000 patients. This concluded that rates of lactic acidosis were low and similar to that of sulphonylureas [26]. A more recent systematic review showed similar results suggesting that risk of lactic acidosis is low and indistinguishable from background risks and that available evidence supported expansion of metformin in patients with CKD with appropriate dose reduction and follow-up [27].

Despite almost 60 years of metformin use, there is a lack of pharmacokinetic studies of metformin in patients with CKD. Metformin inhibits the mitochondrial respiratory chain. This impairs energy metabolism aerobically leading to increased anaerobic metabolism thereby generating lactate [28]. Metformin is eliminated unchanged by the kidney therefore it can accumulate in patients with renal impairment increasing the risk of lactic acidosis. In a small study of 15 patients administered 850 mg of metformin, mild CKD (creatinine clearance, 60-90 mL/min) was associated with 23% to 33% reductions in medication clearance and moderate CKD(30-60 mL/min) with 74% to 78% reductions [29]. Whilst safety of metformin has been shown in patients with mild renal impairment, dose reduction is advisable. Further pharmacokinetic studies are required to establish safe dosing in patients with moderate to severe renal impairment.

It has previously been shown in animal models that metformin exerts a nephrotoxic effect [30]. However, there is a lack of data in humans to support this. A recent retrospective cross sectional study showed that in a small number of patients, with previously normal renal function, admitted with AKI, metformin use was associated with both lactic acidosis and worsening AKI in a dose related manner. They did not, however, demonstrate a difference in survival or need for RRT [31].

There is increasing evidence that AKI is associated with increased mortality even with stage 1 [32–34] AKI. Our data found that survival rates were higher in patients with AKI previously treated with metformin compared to patients with AKI not previously on metformin. This is in line with several previous observational studies in which lower event rates in those exposed to metformin persists even in subgroups with impaired renal function [2, 4]. A Swedish study of 51,675 people with Type 2 diabetes found that those treated with metformin monotherapy had a lower risk of fatal or non-fatal cardiovascular disease events and all-cause mortality [2]. In a multicentre cohort of 19,691 diabetic patients with atherothrombosis, mortality rates were lower in patients treated with metformin compared to those not on metformin. This association persisted in a subgroup of patients with CKD (eGFR 30-60 ml/min/1.73^2^) [4]. A recent review of metformin use in patients with kidney disease recommended that metformin could be used in patients with an eGFR of 30-60 ml/min/1.73^2^ with dose reduction [27]. Following this review the FDA have revised their recommendation stating that it can be used in mild renal impairment and in some with moderate renal impairment [8]. It is difficult to ascertain whether the survival benefit we have shown is due to selection bias in that patients who receive metformin may be a different cohort in that they are fitter. However, we have shown that in our data patients on metformin actually had a longer duration of diabetes and higher HbA_1c_ levels suggesting that the survival benefit may be due to the known beneficial effects of metformin on vascular endothelium rather than selection bias.

Whilst renal safety of metformin is important in its own right, we were also motivated to analyse AKI rates as a proxy for lactic acidosis. It is difficult to ascertain the true incidence of Metformin associated lactic acidosis (MALA). Observational studies may either over or underestimate incidence of MALA [35–37]. Most cases of MALA are multifactorial with other factors contributing to lactic acidosis such as sepsis, cardiac, respiratory or hepatic failure. Studies can be subject to ascertainment bias with fitter patients being selected for treatment with metformin. In addition, patients on metformin are more likely to have lactate levels measured leading to the potential for overestimation of MALA. International Statistical Classification of Diseases and Related Health Problems 10th Revision (ICD −10) coding in isolation cannot be used in observational studies to establish cases of MALA due to a lack of specific code for lactic acidosis and many studies have not included patients with CKD. It is therefore difficult to establish the safety of metformin in patients with CKD. We therefore examined hospitalisations with AKI in patients treated with metformin as clinically significant lactic acidosis occurs in the presence of AKI. Whilst we cannot draw conclusions regarding the rates of MALA in patients with CKD, we have shown that metformin use does not adversely affect mortality which is reassuring. This is arguably more clinically significant than actual rates of MALA.

## Conclusion

Our large cohort of metformin users provides a reassuring message of the safety of metformin in patients with or without a background of CKD and supports the recent revision of FDA and EMA guidance on metformin prescribing in patients with renal impairment. We have also demonstrated that metformin does not adversely affect survival in patients with AKI.

## Abbreviations

AKI: Acute kidney injury
ARB: Angiotensin-II receptor antagonists
ATC: Anatomical therapeutic chemical
CHI: Community health index
CKD: Chronic kidney disease
EMA: European medicines agency
FDA: Food and Drug Administration
HIC: Health informatics centre
IQR: Interquartile range
KDIGO: Kidney disease improving global outcome
MALA: Metformin associated lactic acidosis
MDRD: Modification of diet in renal disease
NHS: National health service
NICE: National Institute for Health and Care Excellence
pyrs: Person years
SCI-DC: Scottish care initiative-diabetes collaboration
SMR01: Scottish morbidity record of hospital admissions
SRR: Scottish renal registry
